# Knowledge, attitude, and practice toward cochlear implants among deaf patients who received cochlear implants

**DOI:** 10.1038/s41598-024-55006-8

**Published:** 2024-02-23

**Authors:** Kunjun Li, Ruhuan Zhou, Wenwen Zheng, Yanbing Zhang, Jianxin Qiu

**Affiliations:** 1 Department of Otorhinolaryngology Head and Neck Surgery, The First Affiliated Hospital of Anhui Medical University, Hefei, 230032 China; 2 Department of Otorhinolaryngology Head and Neck Surgery, The Second People’s Hospital of Hefei, Hefei Hospital Affiliated to Anhui Medical University, Hefei, 230011 Anhui China; 3 Department of Otorhinolaryngology Head and Neck Surgery, Fuyang Hospital of Anhui Medical University, Fuyang, 236112 Anhui China

**Keywords:** Knowledge, attitude, practice, Cochlear implants, Deaf patients, Cross-sectional study, Public health, Health care

## Abstract

Despite the growing use of cochlear implants in deaf patients, there is a lack of data on their knowledge, attitude, and practice (KAP) toward cochlear implants. This study aimed to investigate the KAP toward cochlear implants among deaf patients who received cochlear implants. A web-based cross-sectional study was conducted between August 2022 and December 2022 among deaf patients who had received cochlear implants. A self-administered questionnaire was used to collect demographic characteristics and KAP scores. A total of 526 participants were enrolled; 54.18% were female, 65.40% were above 60 years old, and 61.03% were surveyed at less than 3 years after implantation. The mean knowledge, attitude, and practice scores were 8.15 ± 2.18 (possible range: 0–10), 43.63 ± 6.98 (possible range: 12–60), and 41.11 ± 7.42 (possible range: 11–55), respectively, indicating good knowledge, moderate attitude and practice. Multivariable logistic regression analysis showed that attitude [odd ratio (OR) = 1.24, 95% confidence interval (CI) 1.18–1.29, *P* < 0.001] and unemployment (OR = 0.33, 95% CI 0.17–0.63, *P* = 0.001) were independently associated with practice. Path analysis showed that knowledge directly influenced attitude (*β* = 0.93, 95% CI 0.61–1.19, *P* < 0.001), attitude directly influenced practice (*β* = 0.53, 95% CI 0.46–0.61, *P* < 0.001), and knowledge directly (*β* = 0.77, 95% CI 0.53–1.01, *P* < 0.001) and indirectly (*β* = 0.50, 95% CI 0.34–0.66, *P* < 0.001) influenced practice. Deaf patients who received cochlear implants showed good knowledge, moderate attitude and practice toward cochlear implants. Knowledge should be strengthened to improve attitude and practice toward cochlear implants, which could translate into realistic expectations toward cochlear implants devices and proper care and maintenance.

## Introduction

A cochlear implant is a device that stimulates the spiral ganglion cells of the auditory nerve to restore sensorineural hearing loss^1^. It converts sounds into an electrical signal and delivers it directly to the auditory nerve, bypassing the damaged hearing system^1^. The general indications of cochlear implants include sensorineural hearing loss, > 6 months of age, normal cochlear and cranial nerve VIII, bilateral or unilateral deafness, auditory neuropathy, reliability to follow-up with the medical team, and fit for general anesthesia and surgery^2^. In the United States of America, 5406 and 8509 cochlear implants were placed in 2015 and 2019, respectively^3^. In Europe, about one per 1000 newborns received a cochlear implant between 2010 and 2016^4^. One study from Belgium reported that 78% of deaf children received a cochlear implant, compared with 6.6% of deaf adults^5^.

Cochlear implants are surgically placed by otolaryngologists who work closely with audiologists to make this device effective for patients. This device is arguably the most successful device to replace sensory deprivation. The implant includes an external part (microphone, sound processor, and transmission system) and an internal part (receiver, stimulator, and electrode)^1^. Cochlear implants allow people to hear once deemed irreversibly deaf^1^. Cochlear implants also reverse the social isolation often observed in deaf individuals^6^. Restoring hearing can also extend the patient’s life and prevent the progression of cognitive impairment and dementia^7^.

However, hearing restoration is not perfect, and the device does not perform perfectly in all situations (e.g., noisy background), and they can induce discomfort^8^. They can also lead to socially awkward situations and discrimination^9^. Therefore, the patients receiving such devices must have the proper attitude and expectation regarding their functioning and impact on appearance. In addition, such devices are fragile and must be cared for and maintained properly by the patients to avoid medical expenses or re-implantation; such care requires knowledge and proper practice.

Knowledge, attitude, and practice (KAP) surveys can help identify the elements that could be changed to improve the performance of the studied activity and provide data for designing teaching, training, and continuous education activities^10,11^. A study showed that the KAP toward cochlear implantation was good in parents of children scheduled for implantation^12^. Still, no data are available regarding the KAP toward cochlear implants in adults implanted with such devices. Therefore, this study aimed to evaluate the KAP toward cochlear implants in adult deaf patients after cochlear implantation.

## Methods

### Study design and participants

The cross-sectional study enrolled adult patients after cochlear implantation between August 2022 and December 2022. The inclusion criteria were: (1) > 18 years old; (2) used to be diagnosed with bilateral severe or severe to profound sensorineural hearing loss; (3) received cochlear implantation more than six months; (4) received postoperative speech rehabilitation training; (5) patients who signed the informed consents, were willing to participate in the study and completed the questionnaire. The exclusion criteria were: (1) the questionnaire was not filled in as required, or (2) patients with intellectual disability or cognitive impairment who cannot cooperate to complete the questionnaire. This study was approved by the Ethics Committee of Hefei Second People’s Hospital. Informed consent was obtained before the participants completed the survey.

### Procedures

According to the available guidelines for cochlear implants^13,14^, the self-administered questionnaire was designed by the authors and modified according to the comments of four experts with experience in cochlear implants for multiple years. The pre-test (90 copies) was performed and showed a Cronbach’s *α* of 0.895 and a Kaiser–Meyer–Olkin (KMO) of 0.781, showing high intrinsic consistency.

The final questionnaire included demographic characteristics, and knowledge, attitude, and practice dimensions. The knowledge dimension consisted of 10 items. One point was awarded for correct answers and 0 for incorrect answers or unclear. The total score ranged from 0 to 10 points. The attitude dimension consisted of 12 items scored using a Likert 5-point scale, ranging from very positive (5 points) to very negative (1 point). The total score ranged from 12 to 60 points. The practice dimension consisted of 15 items, of which 11 of them were scored using a Likert five-point scale, ranging from always (5 points) to never (1 point). The total score ranged from 11 to 55 points. A score of more than 80% of the total score was considered “good”, 60%-80% was considered “moderate”, and less than 60% was considered “poor”^15^.

### Questionnaire distribution and quality control

Patients who could be contacted by the researchers and research assistants involved in the study were included through a convenience sampling method. Prior to the study, four research assistants (including one junior physician from the department) were trained online on the methods and precautions for collecting questionnaires and emphasized the importance of authenticity. Online questionnaires were sent to patients via WeChat and administered on the Wenjuanxin platform (https://www.wjx.cn), which generated quick response (QR) codes. Participants logged in and filled out the questionnaire by scanning the QR code. In order to ensure data quality and completeness, each IP address was restricted to one submission, and all questionnaire items were made mandatory. Participants were assured of anonymity while completing the questionnaires. The data collection methods primarily included offline questionnaire administration (when the patients came to the hospital for follow-up or device adjustment), telephone interviews, and online completion through WeChat contact. For offline questionnaire administration, the research assistants helped the patients with the scanning QR code and instructed them how to complete the questionnaire. The research assistants could also hand paper questionnaires to the patients with limited access to technology. Telephone interviews were conducted by a research assistant who first explained the purpose, methodology, and relevant study instructions to the patients and invited them to complete the questionnaire over the phone. Online completion through WeChat contact was the preferred method when possible. The participants had to possess a valid and reliable WeChat ID for logging in, but the WeChat ID was not associated with the questionnaire, and the survey was conducted anonymously. After questionnaire collection, the researchers checked all questionnaires for completeness.

### Statistical analyses

The sample size was calculated using the formula for cross-sectional studies: *α* = 0.05,${\text{n}}={\left(\frac{{Z}_{1-\alpha /2}}{\delta }\right)}^{2}\times p\times \left(1-p\right)$ where ${Z}_{1-\alpha /2}$=1.96 when *α* = 0.05, the assumed degree of variability of *p* = 0.5 maximizes the required sample size, and δ is an admissible error (which was 5% here). The theoretical sample size was 480, which includes an extra 20% to allow for subjects lost during the study.

The statistical analysis software was Stata 17.0 (Stata Corporation, College Station, TX, USA). The continuous data were expressed as mean ± standard deviation (SD) and analyzed using ANOVA. The categorical data were expressed as n (%) and analyzed using the chi-square test. Pearson’s correlation was used to analyze the correlations between the KAP scores. Univariable and multivariable logistic regression analyses were performed for the practice scores. The variables with *P* < 0.05 in the univariable analyses were included in the multivariable analyses; the cut-off for the practice scores was 70% of the data distribution. Path analysis was used to test the following hypotheses: (1) knowledge had impacts on attitude; (2) knowledge had impacts on practice; (3) attitude had impacts on practice. Two-sided *P*-values < 0.05 were considered statistically significant.

### Ethics approval and consent to participate

The study was carried out after the protocol was approved by the Institutional Review Board of The Second People’s Hospital of Hefei Clinical Experiment Ethics Committee. I confirm that all methods were performed in accordance with the relevant guidelines. All procedures were performed in accordance with the ethical standards laid down in the 1964 Declaration of Helsinki and its later amendments.

## Results

### Characteristics of the participants

In this study, 526 participants were enrolled, with the majority residing in Anhui (Supplementary Fig. 1). Among the 526 participants, 54.18% were females, 65.40% were aged over 60 years old, 61.03% had been implanted within 3 years before participation, 61.79% lived in urban areas, 67.30% were married, and 98.48% were covered by medical insurance (Table 1).

**Table 1:** Baseline characteristics and KAP scores.

Variables	n = 526	Knowledge (Mean ± SD)	*P*^a^	Attitude (Mean ± SD)	*P*^b^	Practice (Mean ± SD)	*P*^c^
Total scores		8.15 ± 2.18		43.63 ± 6.98		41.11 ± 7.42	–
Daily use time	11.26 ± 3.50	–		–		–	–
Gender							
Male	241 (45.82)	8.11 ± 2.30	0.859	43.27 ± 7.33	0.123	40.63 ± 7.46	0.094
Female	285 (54.18)	8.18 ± 2.07		43.93 ± 6.66		41.52 ± 7.38	
Age of implantation	39.86 ± 14.02						
< 30	140 (26.62)	7.99 ± 2.28	0.217	43.19 ± 6.55	0.746	40.06 ± 7.31	0.043
30–60	42 (7.98)	8.13 ± 2.20		43.66 ± 7.05		41.33 ± 7.41	
> 60	344 (65.40)	8.79 ± 1.37		44.88 ± 7.74		42.90 ± 7.56	
Duration of implantation (years)							
< 3	321 (61.03)	8.29 ± 1.97	0.301	43.42 ± 6.86	0.397	41.42 ± 7.41	0.220
≥ 3	205 (38.97)	7.92 ± 2.45		43.96 ± 7.17		40.63 ± 7.44	
Place of residence							
Non-urban	201 (38.21)	8.77 ± 1.75	< 0.001	44.65 ± 7.06	0.016	41.46 ± 7.55	0.274
Urban	325 (61.79)	7.76 ± 2.32		43.00 ± 6.86		40.90 ± 7.34	
Education level							
Primary school or below	112 (21.29)	7.13 ± 2.48	< 0.001	42.50 ± 6.55	0.003	40.19 ± 7.83	0.463
Junior high school	157 (29.85)	8.27 ± 1.83		42.45 ± 6.99		41.41 ± 6.34	
High school/technical secondary school	128 (24.33)	8.25 ± 2.28		44.70 ± 7.14		41.14 ± 8.34	
Junior college or above	129 (24.52)	8.78 ± 1.87		44.99 ± 6.82		41.53 ± 7.34	
Current status of work							
Employment	157 (29.85)	8.69 ± 1.88	< 0.001	44.37 ± 7.61	0.471	40.43 ± 7.31	0.092
Unemployment	71 (13.50)	7.94 ± 2.13		42.04 ± 6.64		40.83 ± 8.45	
Retired	42 (7.98)	9.00 ± 2.02		44.48 ± 7.38		43.88 ± 6.54	
Self-employed	21 (3.99)	8.86 ± 1.11		43.43 ± 6.71		42.57 ± 6.86	
Farmer	83 (15.78)	7.34 ± 2.42		43.64 ± 4.86		40.30 ± 6.51	
Other	152 (28.90)	7.79 ± 2.27		43.39 ± 7.31		41.43 ± 7.69	
Income, yuan							
< 2000	178 (33.84)	7.79 ± 2.47	0.167	42.42 ± 6.62	0.046	41.09 ± 7.52	0.980
2001–5000	232 (44.11)	8.27 ± 1.99		44.27 ± 7.26		41.06 ± 7.30	
5001–10,000	71 (13.50)	8.39 ± 2.28		44.79 ± 7.15		41.03 ± 7.96	
> 10,000	45 (8.56)	8.56 ± 1.42		43.31 ± 6.05		41.60 ± 7.01	
Marital status							
Unmarried	116 (22.05)	7.59 ± 2.77	0.208	42.96 ± 6.97	0.227	39.67 ± 7.48	0.029
Married	354 (67.30)	8.29 ± 2.00		43.76 ± 7.16		41.73 ± 7.20	
Widowed	56 (10.65)	8.41 ± 1.59		44.20 ± 5.71		40.18 ± 8.29	
Type of medical insurance (multiple choices)							
Basic medical insurance for urban workers	159 (30.23)	8.66 ± 1.99	< 0.001	44.16 ± 6.63	0.240	41.70 ± 7.66	0.108
New Rural Cooperative Medical Insurance	247 (46.96)	7.96 ± 2.21	0.030	43.75 ± 7.00	0.705	41.73 ± 7.33	0.070
Basic medical insurance for urban residents	121 (23.00)	8.31 ± 1.57	0.434	44.58 ± 7.21	0.098	41.24 ± 7.67	0.987
Medical Insurance for Retired Cadres-	2 (0.38)	10.00 ± 0.00	0.094	51.00 ± 0.00	0.081	51.00 ± 0.00	0.049
Business Insurance-	8 (1.52)	9.13 ± 0.99	0.237	48.38 ± 2.97	0.013	45.00 ± 6.37	0.119
None	50 (9.51)	6.22 ± 2.84	< 0.001	39.78 ± 6.19	< 0.001	37.74 ± 6.51	< 0.001

^a^refers to the comparison of knowledge scores between different demographic characteristics; ^b^ refers to the comparison of attitude scores between different demographic characteristics; ^c^ refers to the comparison of practice scores between different demographic characteristics.

### Knowledge

The mean knowledge score was 8.15 ± 2.18 (possible range: 0–10), indicating good knowledge. Higher knowledge scores were observed in non-urban dwellers (P < 0.001), those with higher education (*P* < 0.001), and those with employment, retired, or freelancing (*P* < 0.001). There were no significant differences in the knowledge scores between different age (*P* = 0.217) or status of implant use (*P* = 0.055) (Table 1). The item with the highest score was K4 (“Cochlear implant devices include implants in the body and speech processors out of the body”, 93.54%), while the item with the lowest rate of correct answers was K7 (“There is no need to carry out scientific and effective auditory speech rehabilitation training after surgery, and speech comprehension, speech expression and language use ability will be naturally restored”, 18.44%) (Table 2).

**Table 2:** Knowledge, attitude, and practice.

Knowledge			True	False	Unclear
K1. There are many causes of deafness, such as viral infection, ototoxic drugs, knocking, genetic and presbycusis deafness, etc. (True)			432 (82.13)	12 (2.28)	82 (15.59)
K2. Hearing loss can lead to a serious decline in the patient’s physical and mental health, quality of life, and social communication skills. (True)			481 (91.44)	6 (1.14)	39 (7.41)
K3. Cochlear implant converts sound signals into electrical signals to stimulate the cochlear nerves and make patients hear through coding strategies. (True)			455 (86.50)	11 (2.09)	60 (11.41)
K4. Cochlear implant devices include implants in the body and speech processors out of the body. (True)			492 (93.54)	0 (0.00)	34 (6.46)
K5. The external part of the cochlear implant is not affected by the surrounding temperature, humidity, or electromagnetic and physical forces. (False)			333 (63.31)	118 (22.43)	75 (14.26)
K6. The postoperative complications include dizziness, facial muscle twitching, or pain. (True)			340 (64.64)	57 (10.84)	129 (24.52)
K7. There is no need to carry out scientific and effective auditory speech rehabilitation training after surgery, and speech comprehension, speech expression, and language use ability will be naturally restored. (False)			376 (71.48)	97 (18.44)	53 (10.08)
K8. The most important thing about the implanted parts in the body is to prevent violent impact and squeeze of the part and avoid displacing the implanted parts in the body. (True)			465 (88.40)	12 (2.28)	49 (9.32)
K9. Keep the appearance and key parts of the cochlear processor clean at all times, clean the microphone radio port, and monitor the headphone jack and other places where dust easily accumulates every day. (True)			485 (92.21)	8 (1.52)	33 (6.27)
K10. Sudden hearing loss should be seen in time. If the treatment time is missed, hearing cannot be rescued. (True)			426 (80.99)	25 (4.75)	75 (14.26)

Attitude	Extremely positive	Positive	Neutral	Negative	Extremely negative
Postoperatively, how satisfied are you with the following health-related quality of life:					
A1. Physiological function:					
A1.1 Basic sound perception (e.g., hearing ambient sounds)	129 (24.52)	227 (43.16)	153 (29.09)	9 (1.71)	8 (1.52)
A1.2 Advanced sound perception (e.g., sound in noisy environments)	74 (14.07)	125 (23.76)	212 (40.30)	95 (18.06)	20 (3.80)
A1.3 Speech ability	115 (21.86)	179 (34.03)	196 (37.26)	25 (4.75)	11 (2.09)
A2. Mental function: self-confidence	115 (21.86)	187 (35.55)	197 (37.45)	16 (3.04)	11 (2.09)
A3. Social function:					
A3.1 Activity (e.g., hearing a car approaching and avoiding it)	178 (33.84)	205 (38.97)	127 (24.14)	11 (2.09)	5 (0.95)
A3.2 Social interaction	78 (14.83)	164 (31.18)	222 (42.21)	54 (10.27)	8 (1.52)
The following statement, to the extent that you endorse is:					
A4. Wearing an electronic cochlear makes me feel inferior	24 (4.56)	70 (13.31)	164 (31.18)	175 (33.27)	93 (17.68)
A5. I would like to wear a cochlear implant every day	227 (43.16)	211 (40.11)	74 (14.07)	9 (1.71)	5 (0.95)
A6. When wearing cochlear electronics, people around me often look sideways, which makes me feel embarrassed	24 (4.56)	85 (16.16)	199 (37.83)	134 (25.48)	84 (15.97)
A7. After the operation, my family and friends took great care of me, making me quickly adapt to wearing cochlear electronics	145 (27.57)	235 (44.68)	135 (25.67)	9 (1.71)	2 (0.38)
A8. Wearing cochlear electronics restricts my daily activities and makes me feel very inconvenient	26 (4.94)	74 (14.07)	218 (41.44)	151 (28.71)	57 (10.84)
A9. Wearing an electronic cochlear should pay attention to the daily environment, which restricts my travel	26 (4.94)	71 (13.50)	185 (35.17)	190 (36.12)	54 (10.27)

Practice	Always	Usually	Sometimes	Occasionally	Never
P1. The frequency of the following situations during your wearing is:					
P1.1 In vitro device failure	7 (1.33)	60 (11.41)	133 (25.29)	184 (34.98)	142 (27.00)
P1.2 Vertigo	16 (3.04)	44 (8.37)	108 (20.53)	193 (36.69)	165 (31.37)
P1.3 Noise	41 (7.79)	103 (19.58)	170 (32.32)	160 (30.42)	52 (9.89)
P1.4 Headache	14 (2.66)	44 (8.37)	110 (20.91)	159 (30.23)	199 (37.83)
P1.5 Hemifacial twitching	9 (1.71)	22 (4.18)	27 (5.13)	102 (19.39)	366 (69.58)
P2. The frequency of the following maintenance behaviors you can do is:					
P2.1 Cleaning	86 (16.35)	217 (41.25)	125 (23.76)	81 (15.40)	17 (3.23)
P2.2 Moisture-proof	151 (28.71)	216 (41.06)	111 (21.10)	35 (6.65)	13 (2.47)
P2.3 Fall resistance	198 (37.64)	120 (22.81)	74 (14.07)	98 (18.63)	36 (6.84)
P2.4 Anti-high temperature	157 (29.85)	156 (29.66)	127 (24.14)	50 (9.51)	36 (6.84)
P2.5 Anti-static	145 (27.57)	154 (29.28)	123 (23.38)	55 (10.46)	49 (9.32)
P2.6 Anti-chemical corrosion	194 (36.88)	145 (27.57)	77 (14.64)	40 (7.60)	70 (13.31)
P3. Whether you have deprecated cochlear electronics?					
Yes	4 (0.76)				
No	522 (99.24)				
P3.1 If deprecated, for what? (Multiple selections)					
Poor effect	–				
Adverse effect	1 (25.00)				
Inconvenient communication	–				
Unaccustomedness	2 (50.00)				
Others	1 (25.00)				

	Disappear completely	Significantly decrease	Slightly decrease	No decrease	Aggravated
P4. If there is tinnitus before surgery, how much does tinnitus decrease after surgery? (if not, please ignore it)	16.09	33.78	27.61	13.14	0
P5. If there is vertigo before surgery, how much does vertigo decrease after surgery? (if not, please ignore it)	24.42	31.4	24.81	13.18	6.2

### Attitude

The mean attitude score was 43.63 ± 6.98 (possible range: 12–60), indicating moderate attitude. There were significant differences in attitude between different residence (*P* = 0.016), education level (*P* = 0.003), income (*P* = 0.046), and medical insurance (*P* < 0.05) (Table 1). Table 2 presents the distribution of the attitude. The extremely positive/positive rates (or very negative/negative for the negative items) were 67.68% for “Postoperatively, how satisfied are you with the following health-related quality of life: A1: physiological function”, 37.83% for “Postoperatively, how satisfied are you with the following health-related quality of life: A1.2: advanced sound perception”, 55.89 for “Postoperatively, how satisfied are you with the following health-related quality of life: A1.3: speech ability”, 57.41% for “A2: Mental function: self-confidence”, 72.81% for “A3.1: Social function: activity”, 46.01% for “A3.2: Social function: social interaction”, 50.95% for “A4: Wearing an electronic cochlear makes me feel inferior”, 83.27% for “A5: I would like to wear a cochlear implant every day”, 41.45% for “A6: When wearing cochlear electronics, people around me often look sideways, which makes me feel embarrassed”, 72.25% for “A7: After the operation, my family and friends took great care of me, making me quickly adapt to wearing cochlear electronics”, 39.55% for “A8: Wearing cochlear electronics restricts my daily activities and makes me feel very inconvenient”, and 46.39% for “A9: Wearing an electronic cochlear should pay attention to the daily environment, which restricts my travel”.

### Practice

The total practice score was 41.11 ± 7.42 (possible range: 11–55), indicating moderate practice. Higher practice scores were observed in participants > 60 years of age (*P* = 0.043) and married participants (*P* = 0.029) (Table 1). Older participants scored higher for the total practice score (*P* = 0.043) and the P2 item (*P* = 0.022) but not for the P1 score (*P* = 0.408). There was no significant difference in the practice scores between different implant use statuses (all *P* > 0.05) (Supplementary Table 1). The practice behaviors are presented in Table 2. The rates of never/occasionally for the symptoms were 61.98% for “in vitro device failure”, 68.06% for “vertigo”, 40.31% for “noise”, 68.06% for “headache”, and 88.97% for “hemifacial twitching”. The patients responded always/usually for the following behaviors: 57.60% for “cleaning”, 69.77% for “moisture-proof”, 60.45% for “fall resistance”, 59.51% for “anti-high temperature”, 56.85% for “anti-static”, and 64.45% for “anti-chemical corrosion”. Only four participants (0.76%) had deprecated cochlear devices. The reasons for deprecation were adverse effects (n = 1, 25.00%), unaccustomedness (n = 2, 50.00%), and others (n = 1, 25.00%) (Table 2). Most participants reported improvements in tinnitus (77.48%) and vertigo (80.63%) (Table 2).

### Pearson correlation analyses

Pearson correlation analyses showed that the knowledge scores were positively correlated with the attitude (r = 0.291, *P* < 0.001) and practice (r = 0.374, *P* < 0.001) scores. The attitude scores were positively correlated with the practice scores (r = 0.572, *P* < 0.001) (Supplementary Table 1).

### Multivariable logistic regression analyses

The multivariable logistic regression analyses showed that the attitude [odd ratio (OR) = 1.24, 95% confidence interval (CI) 1.18–1.29, *P* < 0.001], and unemployment (OR = 0.33, 95% CI 0.17, 0.63, *P* = 0.001) were independently associated with practice (Table 3).

**Table 3:** Univariable and multivariable logistic regression analyses of practice.

Factors	Univariable logistic regression		Multivariable logistic regression	
	OR (95% CI)	*P*	OR (95% CI)	*P*
Knowledge score	1.27 (1.13, 1.42)	< 0.001	1.05 (0.91, 1.21)	0.470
Attitude score	1.22 (1.17, 1.27)	< 0.001	1.24 (1.18, 1.29)	< 0.001
Daily use time	1.05 (1.00, 1.11)	0.068		
Gender				
Male	Ref			
Female	1.11 (0.76, 1.62)	0.597		
Age of implantation				
< 30	Ref			
30–60	1.55 (0.97, 2.46)	0.067	1.24 (0.70, 2.22)	0.461
> 60	2.75 (1.32, 5.72)	0.007	2.32 (0.92, 5.81)	0.074
Duration of implantation (years)				
< 3	Ref			
≥ 3	0.94 (0.64, 1.39)	0.772		
Place of residence				
Non-urban	Ref			
Urban	1.35 (0.92, 1.98)	0.127		
Education level				
Primary school or below	Ref			
Junior high school	1.17 (0.67, 2.03)	0.581		
High school/technical secondary school	1.47 (0.83, 2.58)	0.185		
Junior college or above	1.16 (0.65, 2.06)	0.610		
Current status of work				
Employment	Ref			
Unemployment	0.60 (0.36, 1.00)	0.051	0.33 (0.17, 0.63)	0.001
Retired	0.94 (0.51, 1.73)	0.852	1.11 (0.53, 2.30)	0.782
Self-employed	2.10 (1.05, 4.21)	0.036	1.57 (0.64, 3.82)	0.321
Farmer	1.05 (0.40, 2.77)	0.920	1.09 (0.34, 3.56)	0.881
Other	0.50 (0.26, 0.95)	0.036	0.43 (0.20, 0.91)	0.027
Income level				
< 2000	Ref			
2001–5000	1.09 (0.71, 1.68)	0.692		
5001–10,000	1.11 (0.60, 2.03)	0.745		
> 10,000	0.96 (0.46, 2.00)	0.908		
Marital status				
Unmarried	Ref			
Married	0.51 (0.30, 0.85)	0.010	0.60 (0.31, 1.18)	0.138
Widowed	0.86 (0.46, 1.61)	0.646	0.88 (0.43, 1.82)	0.738

### Path analysis

**Figure 1:**
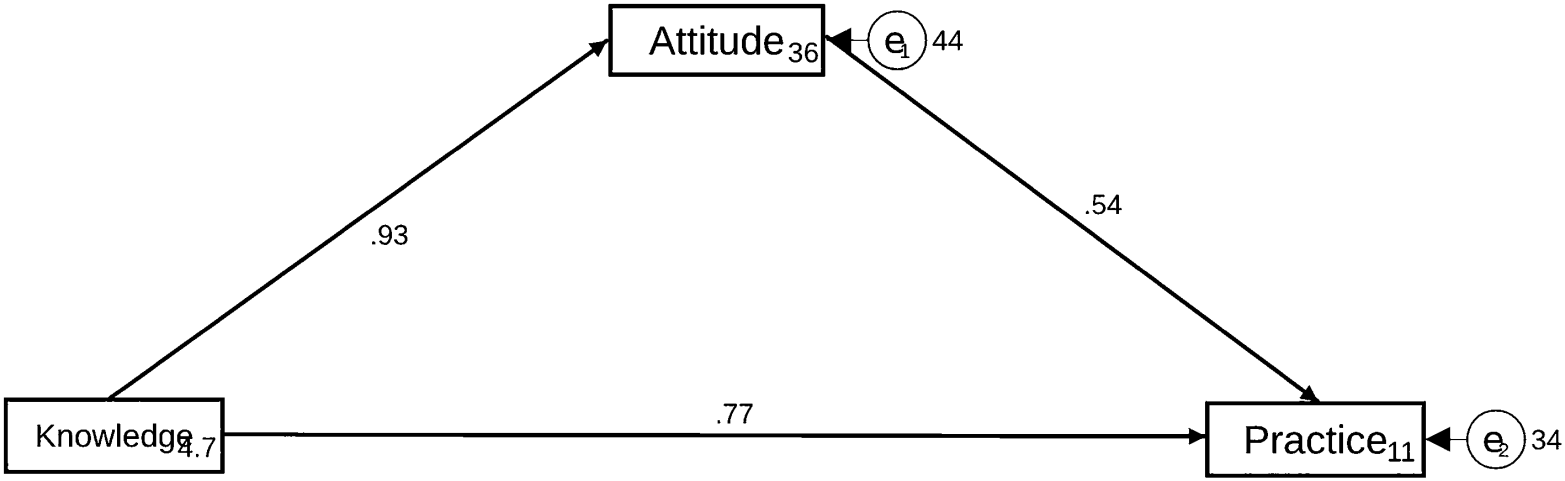
Path analyses.

The path analysis showed that knowledge directly influenced attitude (*β* = 0.93, 95% CI 0.61–1.19, *P* < 0.001), attitude directly influenced practice (*β* = 0.53, 95% CI 0.46–0.61, *P* < 0.001), and knowledge directly (*β* = 0.77, 95% CI 0.53–1.01, *P* < 0.001) and indirectly (*β* = 0.50, 95% CI 0.34–0.66, *P* < 0.001) influenced practice (Fig. 1 and Table 4).

**Table 4:** The direct and indirect estimates of SEM.

Model paths		Total effects		Direct effect		Indirect effect	
		*β* (95% CI)	P_total effect_	*β* (95% CI)	P_direct effect_	*β* (95% CI)	P_indirec effect_
Attitude < -	Knowledge	0.93 (0.67, 1.19)	< 0.001	0.93 (0.67, 1.19)	< 0.001		
Practice < -	Attitude	0.53 (0.46, 0.61)	< 0.001	0.53 (0.46, 0.61)	< 0.001		
Practice < -	Knowledge	1.27 (1.00, 1.54)	< 0.001	0.77 (0.53, 1.01)	< 0.001	0.50 (0.34, 0.66)	< 0.001

## Discussion

This study revealed that the patients had good knowledge and moderate attitude and practice toward cochlear implants. A good attitude was found to have a positive impact on practice. Specific points should be improved and emphasized when teaching the patients about the implants, specifically regarding the care and capabilities of the device.

A previous study in India showed that the parents of children scheduled for cochlear implantation had high KAP regarding cochlear implants^12^, which is unsurprising since it is an invasive procedure with possible complications, and the parents want to be well-informed to ensure the best outcomes for their children. On the other hand, a study in Saudi Arabia showed lower KAP in parents of children with hearing loss^16^. A previous study in patients with hearing loss showed poor knowledge about the eligibility criteria for receiving cochlear implants^17^.

In the present study, the participants were surveyed after implantation and showed good knowledge and active practice but a lower attitude. Still, two important knowledge points would warrant improvements. Indeed, the K5 (“The external part of the cochlear implant is not affected by the surrounding temperature, humidity, electromagnetic and physical forces”) and K7 (“There is no need to carry out scientific and effective auditory speech rehabilitation training after surgery, and speech comprehension, speech expression and language use ability will be naturally restored”) items had low scores. Knowing that the external part is fragile and should be cared for appropriately is essential for ensuring the device’s longevity and avoiding medical expenses due to device replacement. In addition, as the devices evolve, replacement external parts might no longer be compatible with the internal parts, necessitating reoperation. Hence, it is of the utmost importance to stress the good care of the device^18,19^. Not knowing that auditory rehabilitation is necessary after receiving cochlear implants has been reported before and appears to be a common misconception about cochlear implants^20^. Indeed, cochlear implants allow some kind of sound perception, but they do not restore natural hearing^20^. Hence, patients who have never heard will simply learn to hear with the device after implantation. On the other hand, people who have heard naturally for a part of their lives have to relearn how to hear using the novel signals sent by the device^20^. In addition, properly teaching the patients how to take care of their implants should help prevent device damage and medical expenses for replacements.

Importantly, physicians are among the primary sources of reliable medical information for the patients and the population. Still, previous studies showed that the KAP of cochlear implants of otolaryngology and non-otolaryngology physicians could be improved^21–24^. A study in speech and language therapists reported similar results^25^. A study in the United Kingdom suggested that although audiologists had a good knowledge of cochlear implants, they lacked the confidence to discuss the option with their patients^26^. Hence, the literature suggests that the KAP of physicians could be improved, which could help them relay precise information to the patients since physicians are often the primary source of reliable health knowledge. The physicians were not surveyed in the present study, and their KAP in the study area remains unknown. Future studies could address that issue and see how their KAP could be improved.

The attitude questions covered areas about satisfaction with the device and perceived functions related to self-confidence, social function, social perception, and activities of daily living. In the present study, the lower attitude scores were mainly related to hearing expectations relative to the device (e.g., hearing sounds in a noisy environment) and social situations (feeling of inferiority and shyness of being observed in public). Indeed, patients might have misconceptions about what the cochlear implants can do^27,28^. Zeitler & Holcomb^27^ reported five common myths about cochlear implants, the fourth myth being that cochlear implants can restore hearing to “normal”, which is false. For now, the available devices transmit all sounds equally and do not discriminate between background noise and targeted sounds^27,28^. Devices that suppress background noise are being researched but are not yet commercially available^29^. In addition, the device’s external part is obvious, cannot be hidden, and will bring attention to the wearer. Therefore, based on the present study and the known myths about cochlear implants, it appears important to manage the patient’s expectations when discussing the management options for deafness. Therefore, the moderate attitude scores indicate that managing the expectations of the patients should help the patient’s quality of life after surgery and have a better attitude after the surgery.

This study showed that attitude was independently associated with good practice. The SEM analyses also showed that knowledge directly influenced attitude and practice and indirectly influenced practice, and attitude directly influenced practice. Therefore, based on the KAP theory, cultivating a favorable attitude would be conducive to improving the practice^10,11^, including proper cochlear implant care and maintenance. In addition, unemployment and other work status (i.e., other than employment, retired, self-employed, and farmer) were associated with poor practice toward cochlear implants. It could be due to financial or insurance reasons, little time available, and improper/difficult access to healthcare resources. Money and a lack of insurance are the main reasons preventing access to cochlear implants in the United States of America^18^. Programs should be implemented to provide access to cochlear implants to anyone eligible.

Cochlear implants are delicate and expensive devices that require proper care. In the present study, only four participants (< 1%) had deprecated cochlear devices, indicating that most participants were taking proper care of their implants. The exact reasons for taking good care of their devices were not explored in the present study but could be related to the fear of additional medical expenses and living without a cochlear implant. Besides hearing, the improvements in tinnitus and vertigo observed in the present study could be another reason for taking good care of the devices. Indeed, it has been reported that cochlear implants can help reduce tinnitus and vertigo^30–32^. Auditory deprivation is thought to be a major cause of tinnitus^33^. Therefore, restoring hearing using cochlear implants has decreased tinnitus^34^, as reported in the present study. The mechanisms of vertigo improvement are still unknown, but vertigo improvement after cochlear implants has been reported^30,35,36^, as also observed here.

A strength of the present study is the enrollment of a relatively large sample of patients with cochlear implants. It was possible because the authors’ hospital is a tertiary center specializing in such devices. Hence, since the study examined the KAP of the hospital’s population, the results will be directly translated to improve the conditions of the patients.

Nevertheless, this study had limitations. The sample size was relatively small and from a limited geographical area. The study included participants mainly from Anhui province in China, which may restrict the applicability of the findings to other regions or countries with different healthcare systems or patient populations. The questionnaire was designed by the authors and influenced by the local guidelines and practice, limiting generalizability. Furthermore, it was a self-administered questionnaire, introducing potential response bias. In addition, the understanding of the questions can influence the answers. The questions were revised by the experts, who could suggest different formulations, but the understanding of the question was not tested. The use of self-administered questionnaires relies on participants accurately understanding and responding to the questions, which may lead to response bias or misinterpretation of the items. KAP surveys are only a picture of a specific subject at a specific time^10,11^. There is a potential selection bias due to the convenience sampling method. Indeed, convenience sampling may introduce bias as participants were selected based on their availability and contactability by the researchers, potentially limiting the generalizability of the results. Finally, as offline investigations could be influenced by researchers' interpretations of information, there is a potential for social desirability bias. Still, the results can provide guidance for teaching and training activities and can be used as a comparator to evaluate the success of the implemented methods in the future.

## Conclusion

In conclusion, the patients who received a cochlear implant show good knowledge, moderate attitude, and proactive practice toward cochlear implants. It could be advantageous for healthcare professionals to educate patients about the potential benefits and drawbacks of cochlear implants and proper usage and care. Future studies should examine educational interventions that could be implemented to improve the KAP of patients toward cochlear implants.

### Supplementary Information

Supplementary Figure 1.

Supplementary Table 1.

## Data Availability

All data generated or analyzed during this study are included in this published article and its supplementary information files.

## References

[1] Krogmann R. J., Al Khalili Y. Cochlear Implants. StatPearls. Treasure Island (FL) ineligible companies. Disclosure: Yasir Al Khalili declares no relevant financial relationships with ineligible companies.2023.

[2] Manrique M., Zubicaray J., Ruiz de Erenchun I., Huarte A., Manrique-Huarte R. [Guidelines for cochlear implant indication in Navarre]. *Proc.**An Sist Sanit Navar*. **38**, 289–296 (2015). 10.23938/ASSN.0078 26486535

[3] Nassiri AM, Marinelli JP, Lohse CM, Carlson ML (2023). Incidence of cochlear implantation among adult candidates in the United States. Otol. Neurotol..

[4] De Raeve L, Archbold S, Lehnhardt-Goriany M, Kemp T (2020). Prevalence of cochlear implants in Europe: trend between 2010 and 2016. Cochlear Implants Int..

[5] De Raeve L (2016). Cochlear implants in Belgium: Prevalence in paediatric and adult cochlear implantation. Eur. Ann. Otorhinolaryngol. Head Neck Dis..

[6] Clark JH (2012). Cochlear implant rehabilitation in older adults: Literature review and proposal of a conceptual framework. J. Am. Geriatr. Soc..

[7] Mosnier I (2018). Long-term cognitive prognosis of profoundly deaf older adults after hearing rehabilitation using cochlear implants. J Am Geriatr Soc..

[8] Huber M (2022). Cochlear implant-specific risks should be considered, when assessing the quality of life of children and adolescents with hearing loss and cochlear implants-not just cochlear implant-specific benefits-perspective. Front. Neurosci..

[9] Schuh M, Bush ML (2021). Defining disparities in cochlear implantation through the social determinants of health. Semin. Hear..

[10] Andrade C, Menon V, Ameen S, Kumar PS (2020). Designing and conducting knowledge, attitude, and practice surveys in psychiatry: Practical guidance. Indian J. Psychol. Med..

[11] World Health Organization. Advocacy, communication and social mobilization for TB control: a guide to developing knowledge, attitude and practice surveys. http://whqlibdoc.who.int/publications/2008/9789241596176_eng.pdf. Accessed November 22, 2022 (2008).

[12] Riddhima U, Ranjan R (2022). Knowledge, attitude and practice about hearing impairment among the parents of children posted for cochlear implantation. Int. Tinnitus J..

[13] Guidelines for working with cochlear implants (2013). *Chin. J. Otolaryngol. Head Neck Surg.* **49**, (2014).

[14] Tsekoura M, Tsepis E, Billis E, Gliatis J (2020). Sarcopenia in patients with chronic obstructive pulmonary disease: A study of prevalence and associated factors in Western Greek population. Lung India Off. Organ Indian Chest Soc...

[15] Lee F, Suryohusodo AA (2022). Knowledge, attitude, and practice assessment toward COVID-19 among communities in East Nusa Tenggara, Indonesia: A cross-sectional study. Front. Public Health.

[16] Aloqaili Y (2019). Knowledge about cochlear implantation: A parental perspective. Cochlear Implants Int..

[17] Manrique M (2018). Survey on the knowledge of cochlear implant indication in the treatment of hearing loss in Spain. Acta Otorrinolaringol. Esp (Engl Ed)..

[18] Nassiri AM, Marinelli JP, Sorkin DL, Carlson ML (2021). Barriers to adult cochlear implant care in the United States: An analysis of health care delivery. Semin. Hear..

[19] National Health Service. What must I avoid now I have a cochlear implant?. https://www.southtees.nhs.uk/services/audiology/north-east-regional-cochlear-implant-programme/adults/living-with-your-cochlear-implant/what-must-i-avoid-now-i-have-a-cochlear-implant/2021.

[20] Moberly AC (2020). Comprehensive auditory rehabilitation in adults receiving cochlear implants: A pilot study. Laryngoscope Investig. Otolaryngol..

[21] Reddy YM, Anjali L, Augustine A, Mary J, Ajay P (2022). Knowledge, attitude and practices regarding cochlear implants among medical doctors other than otolaryngologists: A prospective cross-sectional study. J. Laryngol. Otol..

[22] Zaitoun M, Alqudah S, Nuseir A (2022). Knowledge, attitude, and practice of hearing loss among physicians in Jordan. Am. J. Audiol..

[23] D'Haese PSC, Van Rompaey V, De Bodt M, Van de Heyning P (2018). The knowledge and beliefs regarding practical aspects of cochlear implants: A study of otorhinolaryngologists in a secondary setting in a multi-country study. Cochlear Implants Int..

[24] Mathews MR, Johnson CE, Danhauer JL (2009). Pediatricians' knowledge of, experience with, and comfort levels for cochlear implants in children. Am. J. Audiol..

[25] Okalidou A (2014). Knowledge, experience and practice of SLTs regarding (re)habilitation in deaf children with cochlear implants. Int. J. Pediatr. Otorhinolaryngol..

[26] Chundu S, Buhagiar R (2013). Audiologists' knowledge of cochlear implants and their related referrals to the cochlear implant centre: Pilot study findings from UK. Cochlear Implants Int..

[27] Zeitler D, Holcomb M (2021). 5 common myths of cochlear implants. Hearing J..

[28] Woodson EA, Aaron K, Nguyen-Huynh A, Vargo J, Mowry SE (2021). Mythbusters! the truth about common misconceptions in cochlear implantation. Semin. Hear..

[29] Zhou H, Wang N, Zheng N, Yu G, Meng Q (2020). A new approach for noise suppression in cochlear implants: A single-channel noise reduction algorithm. Front. Neurosci..

[30] Kurz A, Auinger A, Arnoldner C (2020). Long-term vertigo control after cochlear implantation in patients with end-stage Meniere's disease: A retrospective questionnaire-based cross-sectional study. Wien Klin Wochenschr..

[31] Roemer A, Lenarz T, Lesinski-Schiedat A (2017). Cochlear implantation improves hearing and vertigo in patients after removal of vestibular schwannoma. Int. Tinnitus J..

[32] Assouly K (2021). Cochlear implantation for tinnitus in adults with bilateral hearing loss: protocol of a randomised controlled trial. BMJ Open..

[33] Dotan A, Shriki O (2021). Tinnitus-like, "hallucinations" elicited by sensory deprivation in an entropy maximization recurrent neural network. PLoS Comput. Biol..

[34] Ramakers GG, van Zon A, Stegeman I, Grolman W (2015). The effect of cochlear implantation on tinnitus in patients with bilateral hearing loss: A systematic review. Laryngoscope.

[35] McRackan TR (2014). Cochlear implantation in Meniere's disease patients. Otol. Neurotol..

[36] Mick P (2014). Cochlear implantation in patients with advanced Meniere's disease. Otol. Neurotol..

