# Supplementary figures and images for: Knowledge, attitude, and practice toward cochlear implants among deaf patients who received cochlear implants
Source: Sci Rep. 2024 Feb 23;14:4451. doi: 10.1038/s41598-024-55006-8 (PMC10891060; doi:10.1038/s41598-024-55006-8)

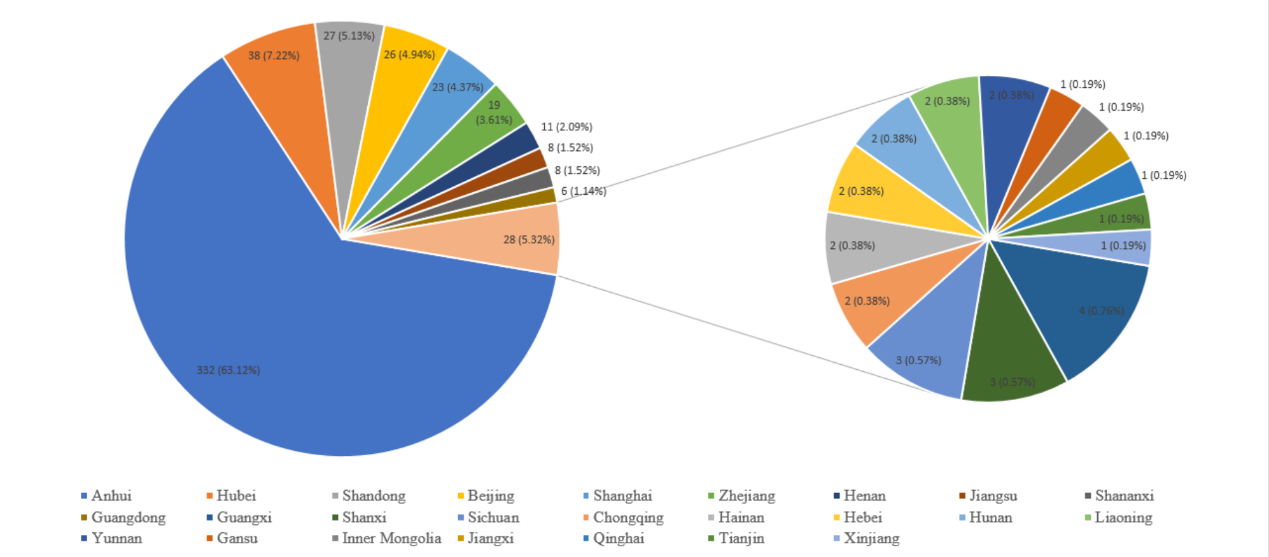


# Supplementary Figure 1. Residence distribution of the participants.

Supplement: Supplementary file 1 — Supplementary Figure 1. [file 41598_2024_55006_MOESM1_ESM.docx]
