# Supplementary material for: Knowledge, attitude, and practice toward cochlear implants among deaf patients who received cochlear implants
Source: Sci Rep. 2024 Feb 23;14:4451. doi: 10.1038/s41598-024-55006-8 (PMC10891060; doi:10.1038/s41598-024-55006-8)
Supplement: Supplementary file 2 — Supplementary Table 1. [file 41598_2024_55006_MOESM2_ESM.docx]

# Supplementary Table 1. Pearson correlation analysis.

|  | Knowledge | Attitude | Practice |
| --- | --- | --- | --- |
| Knowledge | 1 |  |  |
| Attitude | 0.291 (P<0.001) | 1 |  |
| Practice | 0.374 (P<0.001) | 0.572 (P<0.001) | 1 |
